# Identification of mRNAs and lncRNAs Involved in the Regulation of Follicle Development in Goat

**DOI:** 10.3389/fgene.2020.589076

**Published:** 2020-12-17

**Authors:** Zhifeng Zhao, Xian Zou, Tingting Lu, Ming Deng, Yaokun Li, Yongqing Guo, Baoli Sun, Guangbin Liu, Dewu Liu

**Affiliations:** ^1^College of Animal Science, South China Agricultural University, Guangzhou, China; ^2^State Key Laboratory of Livestock and Poultry Breeding, Guangdong Key Laboratory of Animal Breeding and Nutrition, Institute of Animal Science, Guangdong Academy of Agricultural Sciences, Guangzhou, China; ^3^National Engineering Laboratory for Animal Breeding, College of Animal Science and Technology, China Agricultural University, Beijing, China

**Keywords:** RNA-seq, follicle, goat, lncRNA, mRNA

## Abstract

Follicular development and maturation has a significant impact on goat reproductive performance, and it is therefore important to understand the molecular basis of this process. The importance of long non-coding RNAs (lncRNAs) in mammalian reproduction has been established, but little is known about the roles of lncRNAs in different follicular stages, especially in goats. In this study, RNA sequencing (RNA-seq) of large follicles (>10 mm) and small follicles (<3 mm) of Chuanzhong black goats was performed to investigate the regulatory mechanisms of lncRNAs and mRNAs in follicular development and maturation. A total of 8 differentially expressed lncRNAs (DElncRNAs) and 1,799 DEmRNAs were identified, and the majority of these were upregulated in small follicles. *MRO*, *TC2N*, *CDO1*, and *NTRK1* were potentially associated with follicular maturation. KEGG pathway analysis showed that the DEmRNAs involved in ovarian steroidogenesis (*BMP6*, *CYP11A1*, *CYP19A1*, *3BHSD*, *STAR*, *LHCGR*, and *CYP51A1*) and cAMP signaling play roles in regulating follicular maturation and developmental inhibition respectively. Five target pairs of DElncRNA-DEmRNA, namely, ENSCHIT00000001255-*OTX2*, ENSCHIT00000006005-*PEG3*, ENSCHIT00000009455-*PIWIL3*, ENSCHIT00000007977-*POMP*, and ENSCHIT00000000834-*ACTR3* in co-expression analysis provide a clue in follicular development and maturation of lncRNA-mRNA interaction. Our findings provide a valuable resource for lncRNA studies, and could potentially provide a deeper understanding of the genetic basis and molecular mechanisms of goat follicular development and maturation.

## Introduction

In domestic animals such as cattle, sheep, and goats, follicles develop in the form of wave-like pattern during oestrous cycles (Evans, 2003). In each follicular wave, follicles in the early stage grow slowly (Fortune, 1994). However, with the antrum formation, the process speeded up for recruiting qualified follicles to ovulate. Most follicles in follicular wave undergo atresia and only a few dominant follicles develop to ovulate. Many studies have been done on the mechanisms of follicular maturation and atresia. While the question of how developmental follicles go into different fates, stop and turn to atretic or ovulate, still plague researchers.

Follicle development and maturation is regulated by various hormones secreted by the hypothalamus-pituitary-gonadal axis and several growth factors and cytokines expressed by the ovary and follicles. Over the last few decades, it has been reported that a great number of protein-coding genes are involved in the regulation of folliculogenesis, granulosa cell (GC) proliferation, and oocyte maturation (Miller and Auchus, 2011; Chang et al., 2016). For example, activation of epidermal growth factor receptor is required for phosphorylation of SMAD3 by the EGFR-SFKs-ERK1/2 pathway and the subsequent mediation of GC proliferation (Sasseville et al., 2010). Additionally, the estradiol and estrogen receptor system maintains meiotic arrest in oocytes by binding directly to the promoter regions of the *NPPC* and *NPR2* genes (Liu et al., 2017). Resumption of meiosis leads to a decrease in HDAC3 in GCs, and acetylation of histone H3K14 and binding to the SP1 transcription factor to initiate *AREG* transcription and oocyte maturation (Wang et al., 2019). Apart from the protein-encoding genes, many lncRNAs have been found to play roles in follicular cell proliferation and oocyte maturation.

Recent studies have identified several lncRNAs in the ovary that play roles in multiple reproduction events across various species via regulation of protein-coding genes. LncRNA is defined as a non-protein coding transcript with a nucleotide length greater than 200 bp. In general, lncRNAs are characterized by traits such as transcription by RNA polymerase II, alternative splicing, polyadenylation, and capping (Ginger et al., 2006; Mehler and Mattick, 2007; Lagarde et al., 2017). For example, lncRNA-AMH2 was found to activate the expression of *AMH2* in ovarian GCs by enhancing promoter activity (Kimura et al., 2017). Further, in rat polycystic ovarian syndrome, lncRNA-HOTAIR was found to upregulate the expression of *IGF1* and eventually aggravate endocrine disorders and GC apoptosis through competitive binding to miR-130a (Jiang et al., 2020). Additionally, overexpression of another lncRNA, lncRNA-LET, could inhibit migration and promote apoptosis in GCs by upregulating the expression of *TIMP2* and activating the Wnt/β-catenin and Notch signaling pathways (Han et al., 2018). Thus, lncRNAs, along with mRNAs, may be closely involved in the regulation of follicle growth and maturation.

The studies of lncRNAs in human and mice are in progress, which contributed to our understanding of non-coding regulatory mechanisms. However, because of the tissue-specificity of lncRNA expression and low conservation of sequences between species (Derrien et al., 2012; Hezroni et al., 2015; Muret et al., 2019), the roles of lncRNAs in ruminant reproduction, especially in goats, is still poorly understood. The present study aimed to shed light on this question by investigating lncRNA and mRNA expression in follicles of Chuanzhong black goat at different stages and attempting to elucidate the regulatory role of lncRNAs in goat folliculogenesis, providing a theoretical basis for improving ovulation in goat breeding.

## Materials and Methods

### Availability of Supporting Data

This study utilized previously published datasets of 17 samples of uniparous/multiple follicles from 9 Chuanzhong black goats that were classified as 8 large follicles (LF, *d* > 10 mm) and 9 small follicular pools (eight to ten small follicles per pool, SF, *d* < 3 mm) (Zou et al., 2020). The datasets generated and analyzed in the current study are available from the corresponding authors upon request. Sample collection, RNA extraction, preparation of libraries for sequencing, and data processing have been described in a previous study (Zou et al., 2020). After removing the low-quality sequences of adaptor sequences and sequences with quality score <Q20 using Cutadapt, the clean reads obtained were aligned using Bowtie2 (Langmead and Salzberg, 2012) and mapped to the goat reference genome (GCF_001704415.1_ARS1) Ensembl V96 using Tophat2 (Kim et al., 2013). The Q30 (the percentage of the number of bases with phred score >30 in the original data to the total number of bases) of each sample is more than 91%, and is relatively constant within each sample. Moreover, the proportion of clean reads in each library is more than 99%. The proportion of samples located in the reference genome of goat ranged from 83 to 91% (Zou et al., 2020).

### lncRNA Identification

The transcripts were reconstructed using the Stringtie software, and the cuffcompare software was used to identify transcripts in the database that matched the reconstructed transcripts. Candidate lncRNAs were screened from the reconstructed transcripts based on the structural characteristics of the lncRNAs. The inclusion criteria were (1) sequence length of >200 bp, (2) presence of two or more exons, and (3) minimum coverage greater than 3. The CPC, CNCI, and Pfamscan software programs were used to evaluate the protein-coding ability of the candidate lncRNAs (Bateman et al., 2004; Kong et al., 2007; Sun et al., 2013), and transcripts without protein-coding potential in the three programs were considered as novel lncRNAs.

### Expression Analysis

LncRNA and mRNA expression in each sample was evaluated based on the fragments per kilobase per million mapped reads (FPKM). Principal component analysis (PCA), implemented in prcomp function in R, was used to extract the main features of the data as indicators of the overall state of the data and the result was presented by ggplot2 package in R. LncRNAs and mRNAs for which |log_2_Fold-Change| was >1 and FDR was <0.05 were considered to be differentially expressed between small follicles and large follicles, and the prefix “DE” was added so that they are referred to as DElncRNAs and DEmRNAs henceforth. Hierarchical clustering analysis was performed on DElncRNAs and DEmRNAs using the pheatmap package in R^1^.

### Functional Enrichment Analysis

In this study, mRNAs within the nearest upstream or downstream window of the obtained DElncRNAs were included in the *cis*-target mRNA datasets of the DElncRNAs. Gene Ontology (GO)^2^ and Kyoto Encyclopedia of Genes and Genomes (KEGG)^3^ analysis of DEmRNAs and DElncRNAs were performed using the clusterProfiler package in R. Both GO terms and KEGG pathways with FDR < 0.05 were considered to be significantly enriched.

### Construction of DElncRNA–DEmRNA Co-expression Network

Pearson correlation calculations were performed for DEmRNA and DElncRNA (raw *P* value < 0.05) FPKM of 17 samples (8 large follicles and 9 small follicular pools). The correlation pairs for which the absolute value of Pearson correlation coefficient was lower than 0.60 were excluded from the co-localization pairs. The co-expression relationship between DElncRNAs and DEmRNAs was visualized using Cytoscape (V3.5.1) software (Smoot et al., 2011).

### qRT-PCR Verification

The accuracy of RNA-Seq was validated by qRT-PCR by randomly selecting five DElncRNAs and seven DEmRNAs. cDNA for qRT-PCR was synthesized using the PrimeScript RT Reagent Kit with gDNA Eraser (TaKaRa, Dalian, China), and qRT-PCR was performed using the SYBR^®^ Green PCR Supermix (Bio-Rad, CA, United States). Specific primers for the lncRNAs and mRNAs were designed using Primer Premier 5.0 (shown in Table 1). Each 20-μL reaction mixture included 10 μL of SYBR^®^ Green PCR Supermix, 1 μL of each primer, 1 μL of cDNA, and 7 μL of RNase-free H2O. The PCR reaction conditions were as follows: an initial single cycle at 95°C for 1 min, 34 cycles at three different settings (95°C for 30 s, 58°C for 30 s, and 72°C for 1 min), and a final step at 72°C for 1 min. The dissociation curve of amplified products was used to evaluate product specificity. Relative expression levels were normalized to GAPDH with the 2^–ΔΔ*Ct*^ method.

**TABLE 1:** Primer sequences used for qRT-PCR.

mRNA/lncRNA	Primer sequences (5′→3′)	Product size (bp)
*IGFBP5*	F: TGAGACAGGAATCCGAGCAGG R: CGGTCACAGTTGGGCAGGTA	115
*PTCH1*	F: TTCAAGGGTTATGAGTATGTCT R: GGTCGTCGTGGTAAAGGA	153
*TSHR*	F: GGCTCAATTCAACTTTCC	106
	R: ACAACGCTTCTCCTCACT	
*APCDD1*	F: TTCAAGGAATCCCAGTGTCATC R: GTGTTGTTGTGGTAGAATCGGTAG	167
*INHA*	F: TCTCCCAGGCCATCCTTTTTC R: GGGATGGCCGGAACACATAC	105
*KITLG*	F: CATTTATCTTCAACTGCTCCTA R: CCACCATCTCGCTTATCC	191
*MRO*	F: TGTCTTGGAATCTGAGGCT R: TACTTTCTCACCTTGTCCG	157
ENSCHIT00000001721	F: CGTTCTCATGCTGCTTTG R: TATCTTCATTATCGCCACC	180
ENSCHIT00000005825	F: CGTTCTCATGCTGCTTTG R: TATCTTCATTATCGCCACC	128
ENSCHIT00000009619	F: GATTTGTGCTTTGCTGGGTA R: GAGATGAATATGGCTTGTGG	129
ENSCHIT00000005875	F: AAACTCTGCGACTGTGAAATCC R: CCAAAACATCTTTAGCACATCG	106
ENSCHIT00000005031	F: GCTATCACTTCTGCCGACTC R: GGGAATATCTGCTTCACCC	181
MSTRG.11.1	F: CGTGAGGCTGGGAAAGATTGA R: CGGTGGGAGACCTCTGGTTGT	128
*GAPDH*	F: AAGTTCCACGGCACAGTCA R: GGTTCACGCCCATCACAA	247

## Results

### Genomic Features of lncRNAs and mRNAs

**FIGURE 1:**
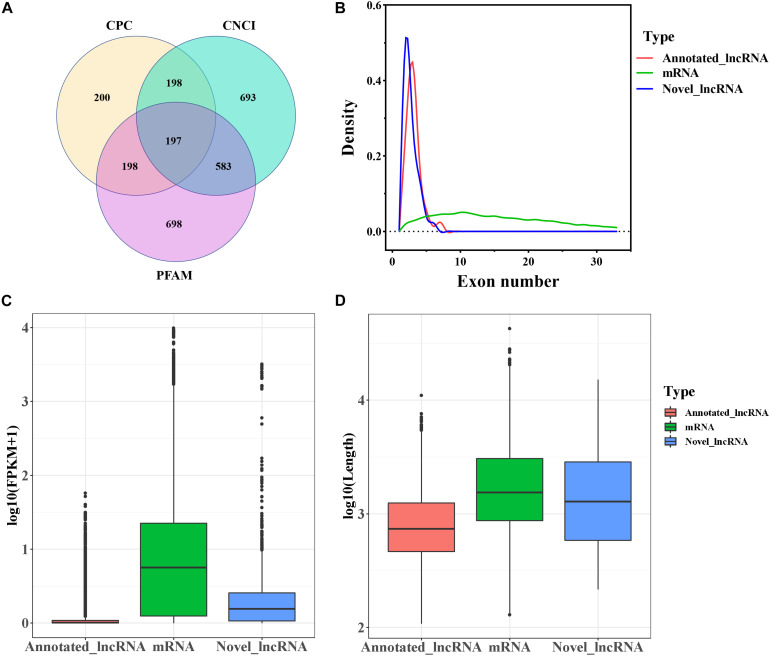
Genomic characteristics of mRNAs and lncRNAs in goat follicles. **(A)** Screening of candidate lncRNAs by CPC, CNCI, and PFAM. **(B)** The density curve of exon numbers. **(C)** Boxplot of the expression level of mRNAs and lncRNAs [expressed as log10 (FPKM+1)]. **(D)** Boxplot of sequence length [expressed as log10 (length)].

In this study, a total of 20,985 mRNAs was detected by RNA-seq. The software programs CPC, CNCI, and Pfam were used to identify novel lncRNAs after removal of the transcripts of protein-coding RNA. Eventually, a total of 4,384 lncRNAs including 197 novel lncRNAs (Figure 1A and Supplementary File 1) were retained. The annotated and novel lncRNAs had fewer exons than mRNAs (Figure 1B), and the expression levels of the annotated and novel lncRNAs were lower than that of mRNAs (Figure 1C). Moreover, the average length of the lncRNAs was lesser than that of the mRNAs (Figure 1D).

### Differentially Expressed lncRNAs and mRNAs

**FIGURE 2:**
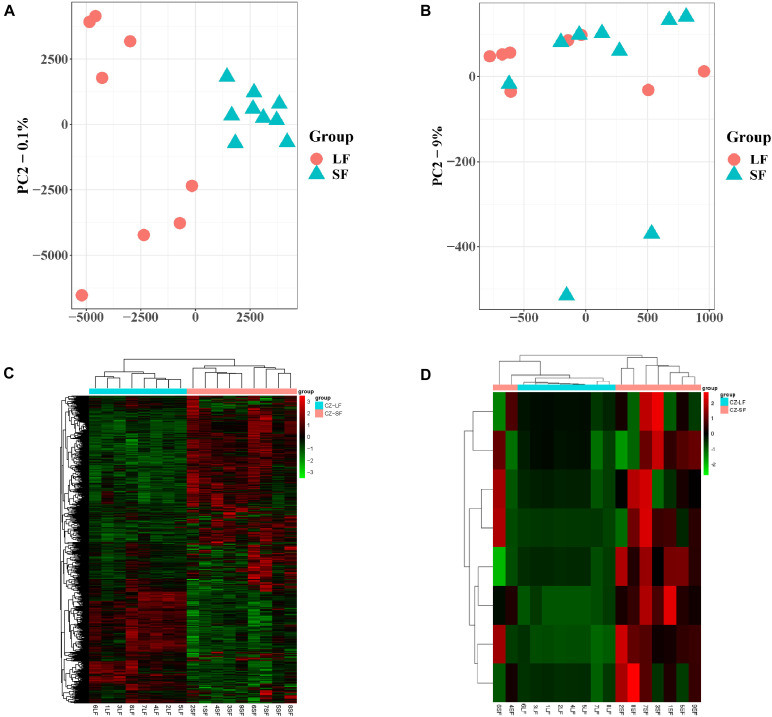
Expression analysis of mRNAs and lncRNAs. **(A)** PCA analysis of mRNA expression with FPKM. **(B)** PCA analysis of lncRNA expression with FPKM. **(C)** Clustering analysis showing the expression profiles of DEmRNAs between SF and LF groups. **(D)** Clustering analysis showing the expression profiles of DElncRNAs between SF and LF groups.

PCA of the mRNA cluster indicated reasonable grouping (Figure 2A), but this was not observed for the lncRNA cluster in small follicles. This is probably because the small follicles in the pools contains different small follicles, which results in the irregular PCA of lncRNAs in small follicular group (Figure 2B). A total of 1,799 mRNAs (1,375upregulated and 424 downregulated mRNAs in small follicles) were differentially expressed between the small and large follicles, based on the criteria applied (|log_2_FoldChange| > 1 and FDR < 0.05 criteria) (Figure 2C and Supplementary File 2). In addition, 8 lncRNAs were found to be up-regulated in small follicles (Figure 2D and Supplementary File 3). The 30 DEmRNAs and 8 DElncRNAs with the most significant differential expression are shown in Table 2.

**TABLE 2:** The 30 DEmRNAs and 8 DElncRNAs with the most significant difference between large follicles and small follicles.

DEmRNAs	Log_2_ Fold-change	FDR	DElncRNAs	Log_2_ Fold-change	FDR
*GLDN*	−3.39	4.34E-14	ENSCHIT00000002664	5.93	6.06E-03
*GLI1*	2.23	6.10E-12	ENSCHIT00000009148	6.10	7.73E-03
*TFR2*	−3.19	6.10E-12	ENSCHIT00000007167	4.41	1.38E-02
*BCL11A*	2.54	6.10E-12	ENSCHIT00000002197	Inf	2.14E-02
*DBI*	−1.97	7.75E-11	ENSCHIT00000008243	5.86	2.31E-02
*ASGR1*	1.92	2.37E-09	ENSCHIT00000002077	5.82	2.58E-02
*MFGE8*	−2.40	4.27E-09	ENSCHIT00000002148	6.07	3.35E-02
*TMEM176B*	−1.75	8.37E-09	ENSCHIT00000010661	Inf	4.30E-02
*GLDC*	2.01	1.82E-08			
*SYCN*	−2.96	1.82E-08			
*KITLG*	−2.49	2.03E-08			
*APCDD1*	2.72	8.38E-07			
*TMEM176A*	−1.56	1.02E-06			
*REG4*	−4.76	1.02E-06			
*TMEM132A*	−2.31	1.02E-06			
*CA14*	4.08	1.02E-06			
*CRISP3*	3.35	2.45E-06			
*ADAM9*	−1.53	2.45E-06			
*TMED6*	−2.14	2.92E-06			
*CD101*	2.04	2.92E-06			
*KLK1*	4.09	3.05E-06			
*INHA*	−3.50	3.89E-06			
*FICD*	−1.88	4.62E-06			
*GUCY2C*	1.69	4.72E-06			
*LOC108633249*	−1.56	7.02E-06			
*ADGRG1*	1.59	7.38E-06			
*FRMPD4*	3.15	8.41E-06			
*FGF10*	1.95	8.75E-06			
*CREB3L3*	1.64	8.75E-06			
*PAPPA*	−5.07	8.94E-06			

### Functional Analysis of DEmRNAs and DElncRNAs

A total of 1,799 DEmRNAs were significantly enriched in 77 GO terms (FDR < 0.05, Supplementary File 4): biological processes, cellular components, and molecular functions account for 22, 33, and 22 of the terms respectively. The mRNAs targeted by DElncRNAs were significantly enriched in three GO terms in cellular components, including basement membrane, proteinaceous extracellular matrix and extracellular matrix component (FDR < 0.05, Supplementary File 4). KEGG pathway analysis of the DEmRNAs revealed 27 enriched pathways among which involved in steroid hormone biosynthesis, steroid biosynthesis, ovarian steroidogenesis, cAMP signaling, and cell adhesion (FDR < 0.05, Supplementary File 5). The pathway, glycosphingolipid biosynthesis ganglio series were the only pathway in KEGG pathway analysis of DElncRNAs (FDR < 0.05, Supplementary File 5). The top 10 GO terms and 27 KEGG pathways for the DEmRNAs are shown in Table 3.

**TABLE 3:** The top 10 GO terms and 27 KEGG pathways for the DEmRNAs.

GO	Description	Count	FDR	Pathway	Count	FDR
Biological	Multicellular organismal process	409	1.06E-05	Retrograde endocannabinoid signaling	31	6.85E-05
processes	Ion transport	103	1.06E-05	Nicotine addiction	14	6.85E-05
	Steroid metabolic process	29	4.87E-04	Drug metabolism – cytochrome P450	17	7.69E-04
	Steroid biosynthetic process	19	6.49E-04	Steroid biosynthesis	8	4.55E-03
	Nervous system development	147	2.88E-03	Protein digestion and absorption	19	4.55E-03
	Cation transport	72	3.09E-03	Terpenoid backbone biosynthesis	8	8.39E-03
	Cholesterol metabolic process	16	3.09E-03	Bile secretion	17	9.23E-03
	Sterol metabolic process	16	3.09E-03	GABAergic synapse	17	9.24E-03
	Regulation of steroid metabolic process	16	3.09E-03	Ovarian steroidogenesis	12	9.24E-03
	Secondary alcohol metabolic process	16	4.56E-03	Steroid hormone biosynthesis	14	1.36E-02
Cellular	Plasma membrane part	152	1.80E-09	Taste transduction	13	3.46E-02
components	Cell periphery	255	2.41E-07	Proximal tubule bicarbonate reclamation	7	3.52E-02
	Membrane part	204	7.44E-07	Taurine and hypotaurine metabolism	5	3.52E-02
	Plasma membrane	244	7.44E-07	Carbohydrate digestion and absorption	10	3.52E-02
	Neuron part	92	1.68E-06	Chemical carcinogenesis	15	3.52E-02
	Integral component of plasma membrane	70	5.03E-06	Salivary secretion	15	3.52E-02
	Intrinsic component of plasma membrane	73	1.01E-05	Renin-angiotensin system	7	3.52E-02
	Membrane	329	1.06E-05	Thyroid hormone synthesis	14	3.55E-02
	Intrinsic component of membrane	107	4.65E-05	ABC transporters	12	3.88E-02
	Integral component of membrane	101	6.68E-05	cAMP signaling pathway	29	3.88E-02
Molecular	Transporter activity	89	2.41E-07	Aldosterone synthesis and secretion	16	4.41E-02
functions	Ion transmembrane transporter activity	68	7.44E-07	Glutathione metabolism	12	4.41E-02
	Transmembrane transporter activity	74	7.44E-07	Synaptic vesicle cycle	12	4.41E-02
	Substrate-specific transmembrane transporter activity	69	3.58E-06	Glutamatergic synapse	18	4.89E-02
	Substrate-specific transporter activity	77	5.27E-06	Cell adhesion molecules (CAMs)	21	4.89E-02
	Cation transmembrane transporter activity	52	4.65E-05	Collecting duct acid secretion	7	4.96E-02
	Ion channel activity	42	5.08E-05			
	Substrate-specific channel activity	42	8.72E-05			
	Channel activity	43	1.28E-04			
	Passive transmembrane transporter activity	43	1.28E-04			

### DElncRNA–DEmRNA Co-expression Network

**FIGURE 3:**
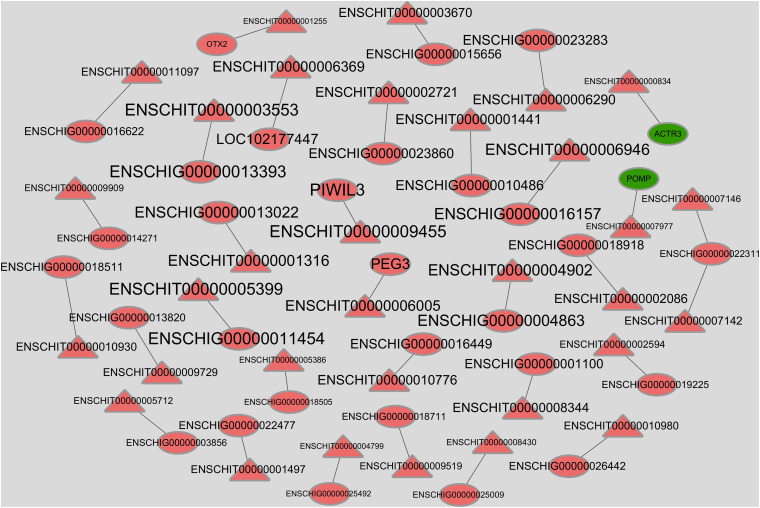
Co-expression network of DElncRNAs and DEmRNAs. The red label represents the up-regulation gene in small follicle, and the green label represents the down-regulation gene in small follicle. The circles represent mRNAs and the triangles represent lncRNAs. The sizes of the labels were arranged according to the absolute value of Pearson correlation coefficient; the larger the value, the larger is the size.

The correlation between co-expressed DEmRNAs and lncRNAs was analyzed using Pearson correlation analysis. Because there may be differences among individuals and the FDR were too large after multiple testing control, we used DElncRNA and mRNA with raw *P* value < 0.05 to perform the co-expression. A total of 32 DEmRNA–lncRNA pairs were found to play a role in follicular development (Figure 3 and Supplementary File 6). Among them, five target pairs, ENSCHIT00000001255-*OTX2*, ENSCHIT00000006005-*PEG3*, ENSCHIT00000009455-*PIWIL3*, ENSCHIT00000007977-*POMP* and ENSCHIT00000000834-*ACTR3* provide a clue in follicular development and maturation of lncRNA-mRNA interaction.

### Validation of RNA-Seq by qRT-PCR

**FIGURE 4:**
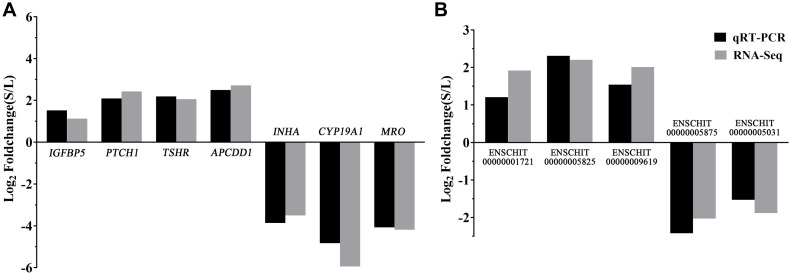
Validation of the DEmRNAs and DElncRNAs by qPCR. **(A)** The qRT-PCR results of the DEmRNAs were compared with the RNA-Seq. **(B)** The qRT-PCR results of the DElncRNAs were compared with the RNA-Seq. Black: qRT-PCR; Gray: RNA-Seq. In each group, 4 samples were analyzed.

To validate the reliability of the RNA-seq results, five DElncRNAs (ENSCHIT00000001721, ENSCHIT00000005825, ENSCHIT00000009619, ENSCHIT00000005875, and ENSCHIT00000005031) and seven DEmRNAs (*IGFBP5*, *PTCH1*, *TSHR*, *APCDD1*, *INHA*, *CYP19A1*, and *MRO*) were randomly selected for qRT-PCR verification. The expression pattern of these DElncRNAs and DEmRNAs was found to be consistent with the patterns determined by RNAs-Seq analysis; therefore, the reliability of the sequencing results was confirmed (Figure 4).

## Discussion

In this study, goat follicle samples deposited in a database were analyzed to identify the mRNAs and lncRNAs that are potentially involved in the regulatory mechanisms underlying follicular development and maturation.

A total of 8 DElncRNAs and 1,799 DEmRNAs were identified by RNA-seq. *KITLG*, *INHA*, *FGF10*, and *PAPPA* were among the 30 most significant DEmRNAs that were found to participate in follicular maturation. These mRNAs have been previously found to play roles in follicle maturation in other species (Driancourt et al., 2000; Rivera and Fortune, 2003; Buratini et al., 2007; Zhang et al., 2010; Namwanje and Brown, 2016). Specifically, 43 DEmRNAs were identified based on the set criteria (FPKM > 10 and |log_2_FoldChange| > 2, Supplementary File 7). In total, we found that 6 of the 43 highly expressed genes (*LHCGR*, *TMEM200A*, *STAR*, *3BHSD*, *CDO1*, and *NTRK1*) were also differentially expressed in the follicles between uniparous and multiple in the earlier paper. They were up-regulated in large follicles compared with small one, and were up-regulated in multiple small follicles compared with uniparous small follicles, suggesting that these genes are not only involved in follicular maturation regulation but also improving the litter size through acting on in small follicles of multiple goats (Zou et al., 2020). Among them, *LHCGR*, *STAR*, and *3BHSD* are known regulatory genes responsible for LH signal acceptance, conversion of cholesterol into pregnenolone, and conversion of progesterone and testosterone in the process of ovarian steroidogenesis which is one of the most important hormonal pathways known for follicular maturation (Miller and Auchus, 2011). In addition, *CDO1*, *NTRK1*, *MRO*, and *TC2N* (the other two genes in the list of 43 genes) were all upregulated in large follicles and might therefore potentially participate in the regulation of follicular maturation in goats, which have not been identified in the context of follicular development and maturation before (as far as we know). The protein encoded by *CDO1* affects mitochondrial function by converting cystine to cysteine sulfamic acid and inhibits the production of glutathione to promote apoptosis, and it has been used as a diagnostic marker in a variety of cancers (Kang et al., 2019; Nishizawa et al., 2019). The NTRK1 protein was found to facilitate follicle assembly and early follicular development in the mouse ovary (Kerr et al., 2009). *MRO* was first found to be expressed in the testis and was associated with testis differentiation, and it is considered as a candidate testis-determining gene (Smith et al., 2003). A recent study had found that *MRO* was also expressed abnormally in ovarian cumulus cells of polycystic ovaries, but its function in follicular maturation is unknown (Kenigsberg et al., 2017). TC2N is a tandem C2 domain-containing protein that was found to attenuate p53 signaling via Cdk5 degradation or disruption of the interaction between Cdk5 and p53, thus inhibiting cell proliferation and, subsequently, tumorigenesis (Hao et al., 2019). In summary, *LHCGR*, *TMEM200A*, *STAR*, *3BHSD*, *CDO1*, and *NTRK1* are not only involved in follicular maturation regulation but also improving the litter size through acting on in small follicles of multiple goats. The underlying mechanism of *CDO1*, *NTRK1*, *MRO*, and *TC2N* in follicular maturation needs to be further investigated.

In the present study, enrichment analysis of the identified DEmRNAs was used to understand the important metabolic and molecular differences between small follicles and large ones in goats. KEGG pathway analysis indicated that ovarian steroidogenesis, steroid biosynthesis, cAMP signaling, and cytokine–cytokine receptor interaction were closely related to follicular development. In particular, ovarian steroidogenesis and steroids biosynthesis were upregulated in large follicles. Ovarian steroidogenesis is one of the most important processes in follicular maturation; therefore, the related mRNAs could be used as important markers for goat follicular maturation. Many mRNAs, such as *BMP6*, *CYP11A1*, *CYP19A1*, *3BHSD*, *STAR*, *LHCGR*, and *CYP51A1*, have been shown to promote follicular development by regulating steroid production in the ovary (Segaloff et al., 1990; Miller and Auchus, 2011; Rajesh et al., 2018). However, 1,375 of the 1,799 DEmRNAs identified in this study were found to be more actively expressed in small follicles. For example, a total of 29 mRNAs were involved in cAMP signaling. cAMP signaling has previously been implicated that it was expressed in mature follicles to maintain oocyte meiosis inhibition in ovarian (Gulappa et al., 2015; Lyga et al., 2016). In particular, the downstream genes of *PTCH1*, *GLI1*, *HHIP*, and *AMH* were found to be upregulated in small follicles, suggesting that these genes are implicated in Hedgehog and AMH transduction in small follicles. These findings might suggest that unselected small follicles have more complicated signal transduction or functions in follicular cells before they undergo apoptosis.

8 different expression lncRNAs, ENSCHIT00000002664, ENSCHIT00000009148, ENSCHIT00000007167, ENSCHIT 00000002197, ENSCHIT00000008243, ENSCHIT00000002077, ENSCHIT00000002148, and ENSCHIT00000010661 were all upregulated in the small follicles. Among them, three DEmRNAs, *NTN1*, *AMIGO2*, and *ENSCHIG00000016389*, were located near ENSCHIT00000007167, ENSCHIT00000002077, and ENSCHIT00000010661, respectively, which involved in the follicular process potentially. Study had shown that *NTN1* could inhibit granulosa cell viability and estradiol 17β levels while it stimulates progesterone production (Basini et al., 2011). The potential core regulating factors, 32 target pairs of DElncRNA–DEmRNA with raw *P* value, were predicted using bioinformatics analysis in our study. 2 of target mRNA with negative correlation, ENSCHIT00000007977-*POMP* and ENSCHIT00000000834- *ACTR3*, were upregulated in large follicles. The rest of 30 target pairs with positive correlation were upregulated in small follicles. Among them, three target pairs, namely, ENSCHIT00000001255-*OTX2*, ENSCHIT00000006005-*PEG3*, and ENSCHIT00000009455-*PIWIL3*, might be involved in follicular development inhibition by transcriptional regulation of genes. In particular, *OTX2* targeted by ENSCHIT00000001255 could affect animal reproductive ability by promoting GnRH promoter activity in GnRH neuron cells, as *OTX2* mutation was associated with hypogonadotropism (Diaczok et al., 2011). *OTX2* has also been found to be differentially expressed in follicles and oocytes, but its regulatory mechanisms remain unclear (Assou et al., 2006; Wu et al., 2016). Thus, the function of the lncRNA ENSCHIT00000001255 might be associated with the function of its target gene *OTX2*. Similarly, ENSCHIT00000006005 might play a role in follicular development inhibition through its target gene *PEG3*. Loss of *PEG3*-imprinted methylation was observed in individual blastocyst stage embryos after *in vitro* oocyte culture and hyperovulation in mice (Song et al., 2009; Market-Velker et al., 2010). Additionally, loss of *PEG3* in a way of promoter methylation might stimulate clonogenic growth and contribute to the pathogenesis of a majority of ovarian cancers, suggesting the potential effect of *PEG3* in female reproduction (Feng et al., 2008). The PIWIL3 protein belongs to the P-element induced wimpy testis-like family, and it plays a role in several stages of genome-wide DNA methylation and meiosis of germ cells (Juliano et al., 2011; Iwasaki et al., 2015). *PIWIL3* has been found to affects oocyte development and embryogenesis (Roovers et al., 2015); therefore, it might play a similar role in goat too. Based on all these findings, it is possible that the lncRNAs ENSCHIT00000001255, ENSCHIT00000006005, and ENSCHIT00000009455 are involved in the regulation of promoter activity or methylation, potentially exerting co-regulatory effects on gene transcription in small follicles.

In the present study, the findings indicate that small follicles in goat might involve a complex mechanism of gene expression in follicular inhibition. Several mRNAs and lncRNAs that potentially play important roles in follicular maturation were identified, based on the expression of the classical protein-coding genes in large follicles. However, we were unable to identify a fairly large number of lncRNAs in the goat gene pool. Accordingly, our future work will focus on verifying the functions of the already identified lncRNAs, so as to provide a richer understanding of the molecular mechanism of goat follicular development and maturation.

In conclusion, the present study provides systematic information about the expression of mRNAs and lncRNAs at different stages of antral follicle in goats. Specifically, DEmRNAs and DElncRNAs involved in important biological processes and pathways associated with follicular development inhibition and maturation were identified, and this provides a valuable resource for lncRNA and mRNA studies of goat follicles.

## Data Availability Statement

The datasets analyzed for this study are publicly available. This data can be found in NCBI SRA (accession code: PRJNA579007).

## Ethics Statement

The animal study was reviewed and approved by Institutional Animal Care and Use Committee of South China Agricultural University (Approval No. 2018-P002).

## Author Contributions

GL and DL designed and managed the project. ZZ, XZ, TL, and MD performed the experiment. YL, YG, and BS carried out the data processing and analysis. ZZ and XZ were major contributors in writing the manuscript. All authors have read and approved the manuscript.

## Conflict of Interest

The authors declare that the research was conducted in the absence of any commercial or financial relationships that could be construed as a potential conflict of interest.

## Footnotes

1 https://cran.r-project.org/web/packages/pheatmap/index.html

2 http://geneontology.org/

3 http://www.kegg.jp/

## Supplementary Material

Supplementary File 1 Novel lncRNAs identified in the goat follicles.

Supplementary File 2 RNA-seq data of DEmRNA expression in large and small follicles.

Supplementary File 3 RNA-seq data of DElncRNA expression in large and small follicles.

Supplementary File 4 Gene ontology analysis of DEmRNAs and DElncRNAs.

Supplementary File 5 Kyoto encyclopedia of genes and genomes analysis of DEmRNAs and DElncRNAs.

Supplementary File 6 The DElncRNA–DEmRNA co-expression network basing on the co-localization (raw *P* value < 0.05).

Supplementary File 7 The subset of 43 DEmRNAs whose FPKM is greater than 10 and absolute value of log_2_FoldChange is larger than 2.

## Abbreviations

lncRNAs: long non-coding RNAs
RNA-seq: RNA sequencing
DElncRNAs: differentially expressed lncRNAs
DEmRNAs: differentially expressed mRNAs
GC: granulosa cell
FPKM: fragments per kilobase per million mapped reads
PCA: principal component analysis
GO: gene ontology
KEGG: Kyoto Encyclopedia of Genes and Genomes.
