# Corrigendum: Identification of mRNAs and lncRNAs Involved in the Regulation of Follicle Development in Goat

**DOI:** 10.3389/fgene.2020.647155

**Published:** 2021-02-02

**Authors:** Zhifeng Zhao, Xian Zou, Tingting Lu, Ming Deng, Yaokun Li, Yongqing Guo, Baoli Sun, Guangbin Liu, Dewu Liu

**Affiliations:** ^1^College of Animal Science, South China Agricultural University, Guangzhou, China; ^2^State Key Laboratory of Livestock and Poultry Breeding, Guangdong Key Laboratory of Animal Breeding and Nutrition, Institute of Animal Science, Guangdong Academy of Agricultural Sciences, Guangzhou, China; ^3^National Engineering Laboratory for Animal Breeding, College of Animal Science and Technology, China Agricultural University, Beijing, China

**Keywords:** RNA-seq, follicle, goat, lncRNA, mRNA

In the published article, there was an error in the affiliation order. Instead of “^1^ College of Animal Science, South China Agricultural University, Guangzhou, China, ^2^ National Engineering Laboratory for Animal Breeding, College of Animal Science and Technology, China Agricultural University, Beijing, China, ^3^ State Key Laboratory of Livestock and Poultry Breeding, Guangdong Key Laboratory of Animal Breeding and Nutrition, Institute of Animal Science, Guangdong Academy of Agricultural Sciences, Guangzhou, China,” it should be “^1^ College of Animal Science, South China Agricultural University, Guangzhou, China, ^2^ State Key Laboratory of Livestock and Poultry Breeding, Guangdong Key Laboratory of Animal Breeding and Nutrition, Institute of Animal Science, Guangdong Academy of Agricultural Sciences, Guangzhou, China, ^3^ National Engineering Laboratory for Animal Breeding, College of Animal Science and Technology, China Agricultural University, Beijing, China”.

Therefore, instead of “Zhifeng Zhao^1,2†^, Xian Zou^3†^” it should be “Zhifeng Zhao^1,3†^, Xian Zou^2†^”.

The authors apologize for this error and state that this does not change the scientific conclusions of the article in any way. The original article has been updated.

